# Gout of the iliopsoas muscle combined with tuberculosis infection causing persistent fever: a case report and literature review

**DOI:** 10.3389/fmed.2025.1601095

**Published:** 2026-01-05

**Authors:** Mei-Ren Zhang, Jian-Hui Hu, Jian-hao Guan, Xiao Zeng, Hai-Yun Chen

**Affiliations:** 1 The Second Clinical College of Guangzhou University of Chinese Medicine, Guangzhou, China; 2 Department of Orthopedics Trauma, Zhuhai Hospital, Guangdong Provincial Hospital of Chinese Medicine, Zhuhai, China

**Keywords:** gout, iliopsoas muscle, tuberculosis, persistent fever, diagnosis

## Abstract

**Background:**

Gout involves the deposition of monosodium urate (MSU) crystals in the body, which can have varied presentations but commonly presents in the peripheral joints. However, gout of the iliopsoas muscle is extremely rare. Moreover, the literature on gout of the iliopsoas muscle combined with tuberculosis (TB) infection—which can mimic common pelvic abscesses—is more limited.

**Case presentation:**

A case of a psoas muscle abscess with a persistently high fever following gout of the iliopsoas muscle, combined with tuberculosis infection, is reported in this study. We present the case of a 71-year-old woman who presented with deep, diffuse pain in the lower back and left hip and a persistently high fever for 1 week. She showed no response to systemic anti-infective treatment. A dual-energy computed tomography (CT) scan showed multiple bilateral gout nodules around the iliac bone, sacrum, and proximal femur. A contrast-enhanced magnetic resonance imaging (MRI) scan revealed a large hyperdense cystic lesion extending along the iliopsoas muscle and erosion and widening of the left sacroiliac joint. The patient received open surgical intervention to achieve effective drainage via a para-rectus approach. Some milky tophi were scraped from the cystic lesion in the iliopsoas muscle. Intraoperative pathology of these tissues confirmed gout formation. High-throughput gene sequencing of these tissues detected various divergent mycobacterium tuberculosis, without evidence of other bacteria, fungi, or anaerobic bacteria. A diagnosis of a pyogenic psoas abscess due to gout of the iliopsoas muscle, combined with tuberculosis infection, was made. The patient responded well to the therapy and had an uncomplicated recovery after anti-gout and anti-tuberculosis treatment.

**Conclusion:**

The development of an iliopsoas abscess as a consequence of gout in the iliopsoas muscle combined with tuberculosis infection is rare. Making a diagnosis in such an unusual case can be challenging. For patients with unexplained high fever as the main clinical symptom, systemic anti-infective treatment alone may not be effective. High-throughput gene sequencing for various pathogens is very helpful in identifying the cause of the pathogen. Open surgical intervention using a para-rectus approach for effective drainage is highly effective and a routine procedure.

## Background

Gout is the most common crystal-induced arthritis. Gouty tophi typically deposit in the extremities, particularly around peripheral joints such as toes and fingers (1). Deposits can have varied presentations, often occurring in peripheral regions where the temperature is lower, such as in the joint capsule, synovial membrane, cartilage, and bone (2, 3). To our knowledge, gout of the iliopsoas muscle is extremely rare, with only one known case in the English literature (1) and one case in Chinese literature (4). In this study, we report an unusual case of a psoas muscle abscess with a persistently high fever following gout of the iliopsoas muscle, combined with tuberculosis infection.

## Case presentation

**Figure 1:**
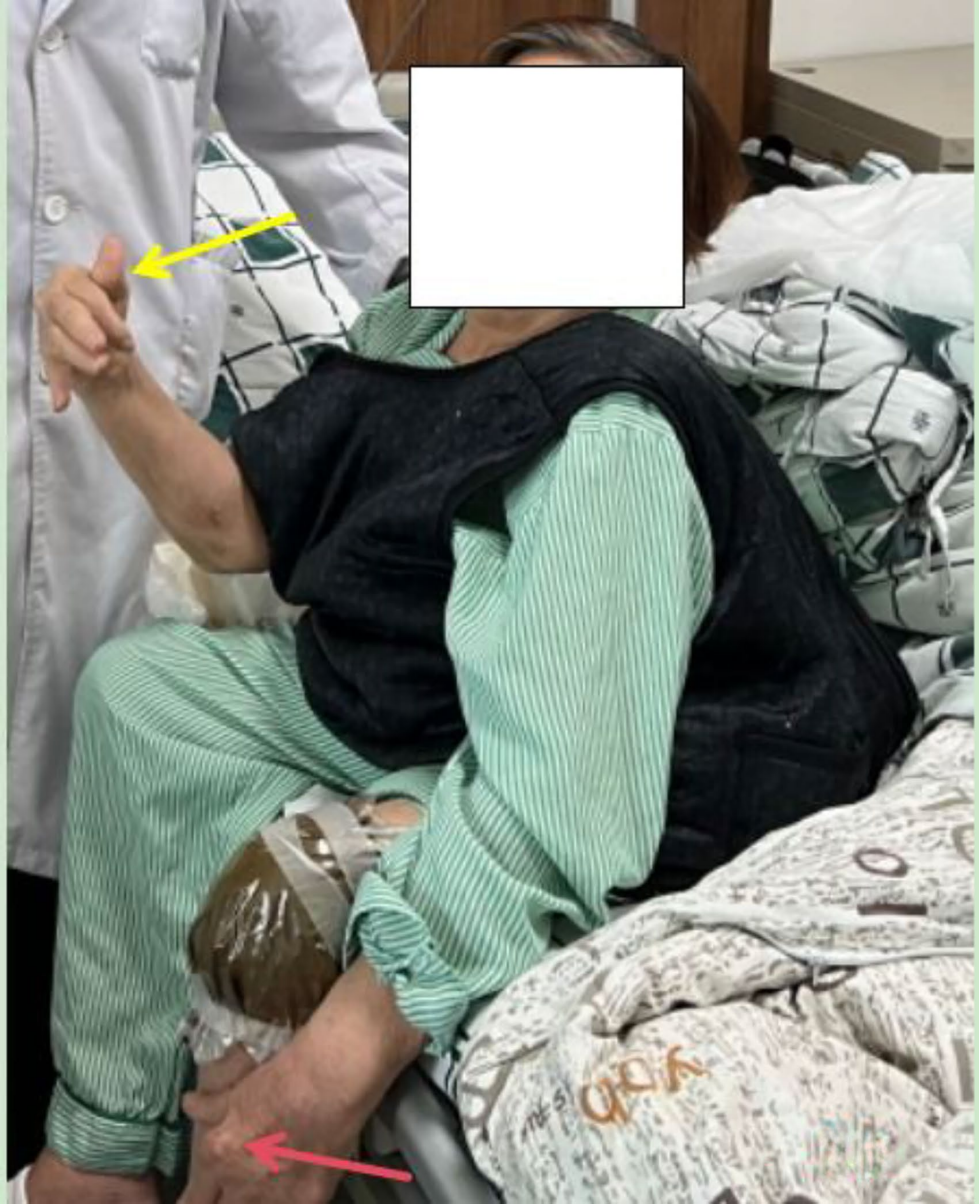
A-P view of patient after admission showed an obviously deformities of right hands (yellow arrow) and tophi formation over left finger (red arrow).

**Figure 2:**
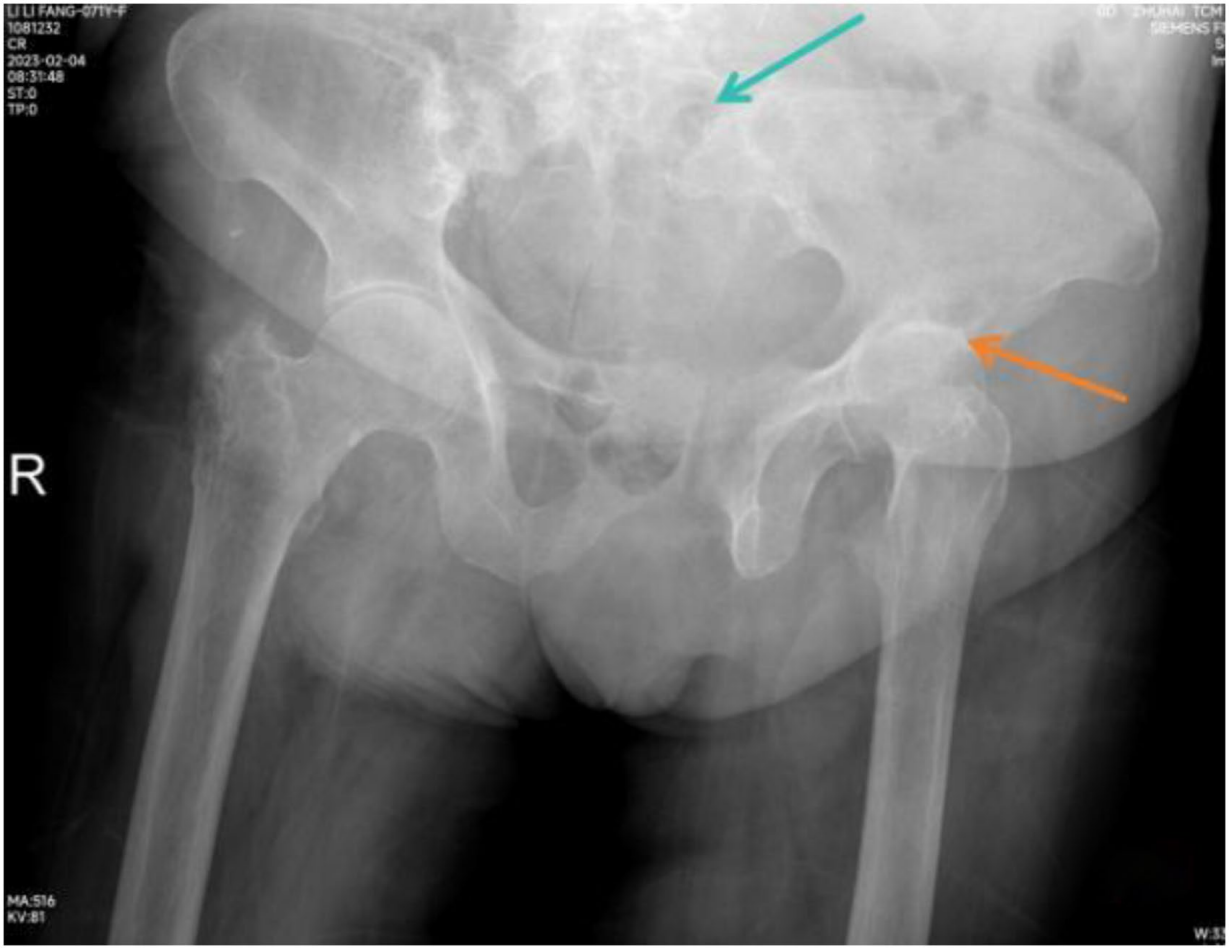
Plain pelvic radiographs showed erosion of the sacral and iliac borders with widening of the left sacroiliac joint (chrysanthemum blue arrow), space narrowing, low-density cystic lesion of left hip (orange arrow).

**Figure 3:**
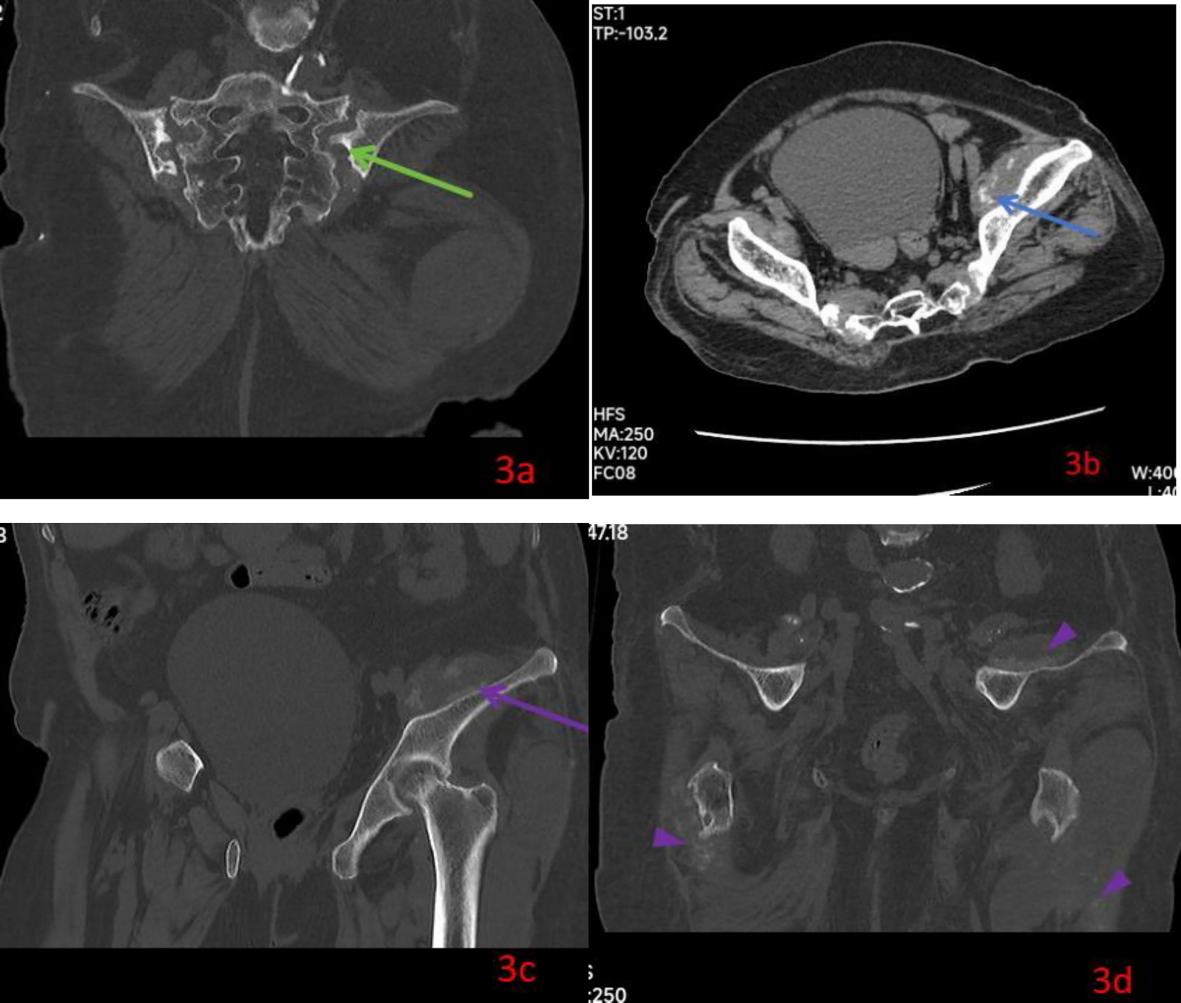
Coronal view of pelvis CT **(a)** after admission showed erosion of the sacral and iliac borders with widening of the left sacroiliac joint and condensation of the adjacent bone green arrow; Horizontal view **(b)** of pelvis CT showed a multilocular abscess within the left psoas muscle (blue arrow); Coronal view of pelvis CT **(c,d)** showed a multilocular abscess within the left psoas muscle (purple arrow) and juxta-articular soft tissue masses containing sandy calcification around pelvis and both hip (purple triangle).

**Figure 4:**
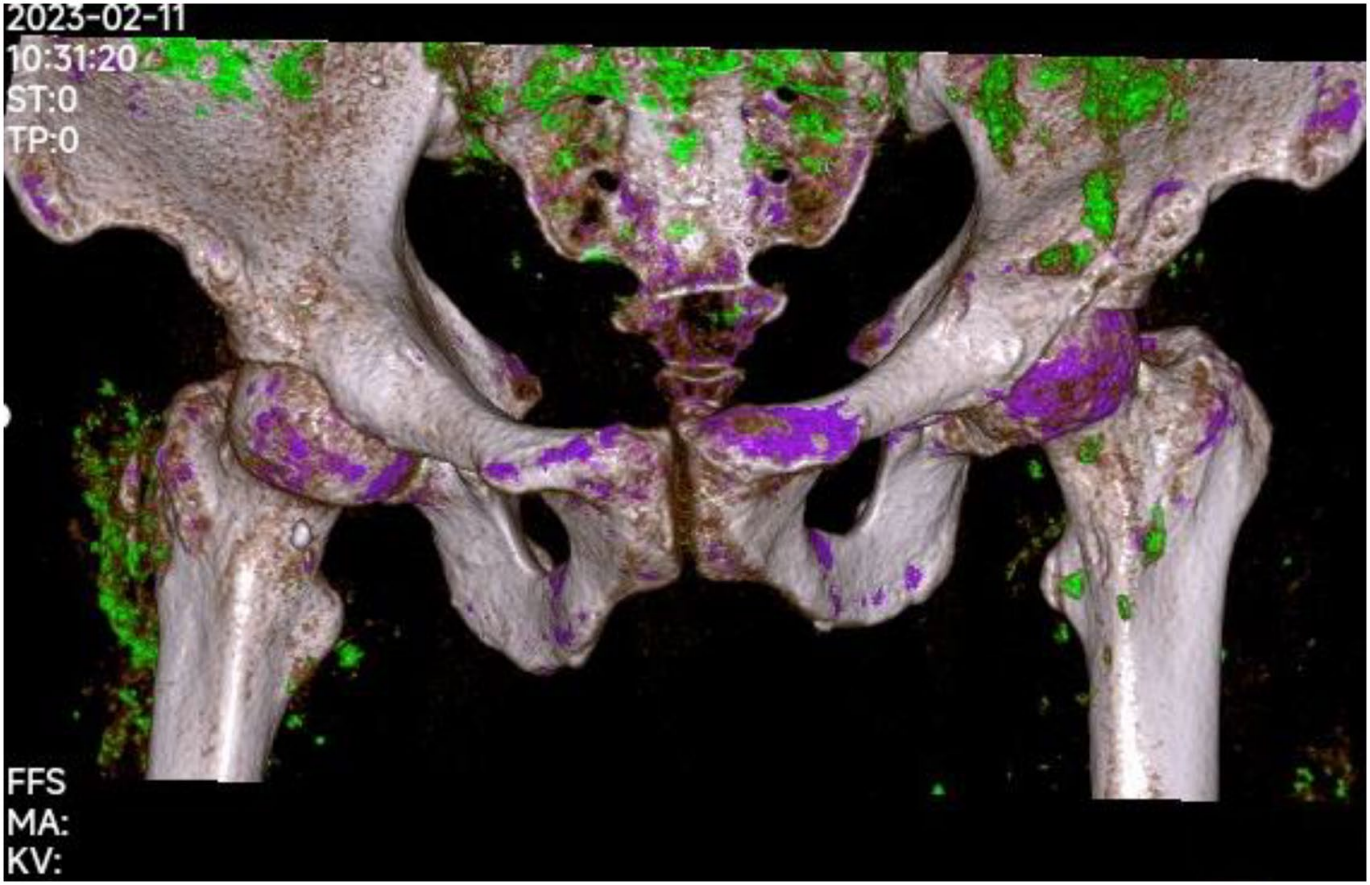
Dual-energy CT scan of pelvic 9 days after admission showed multiple gout nodules around bilateral iliac bone and sacrum and the proximal femur.

**Figure 5:**
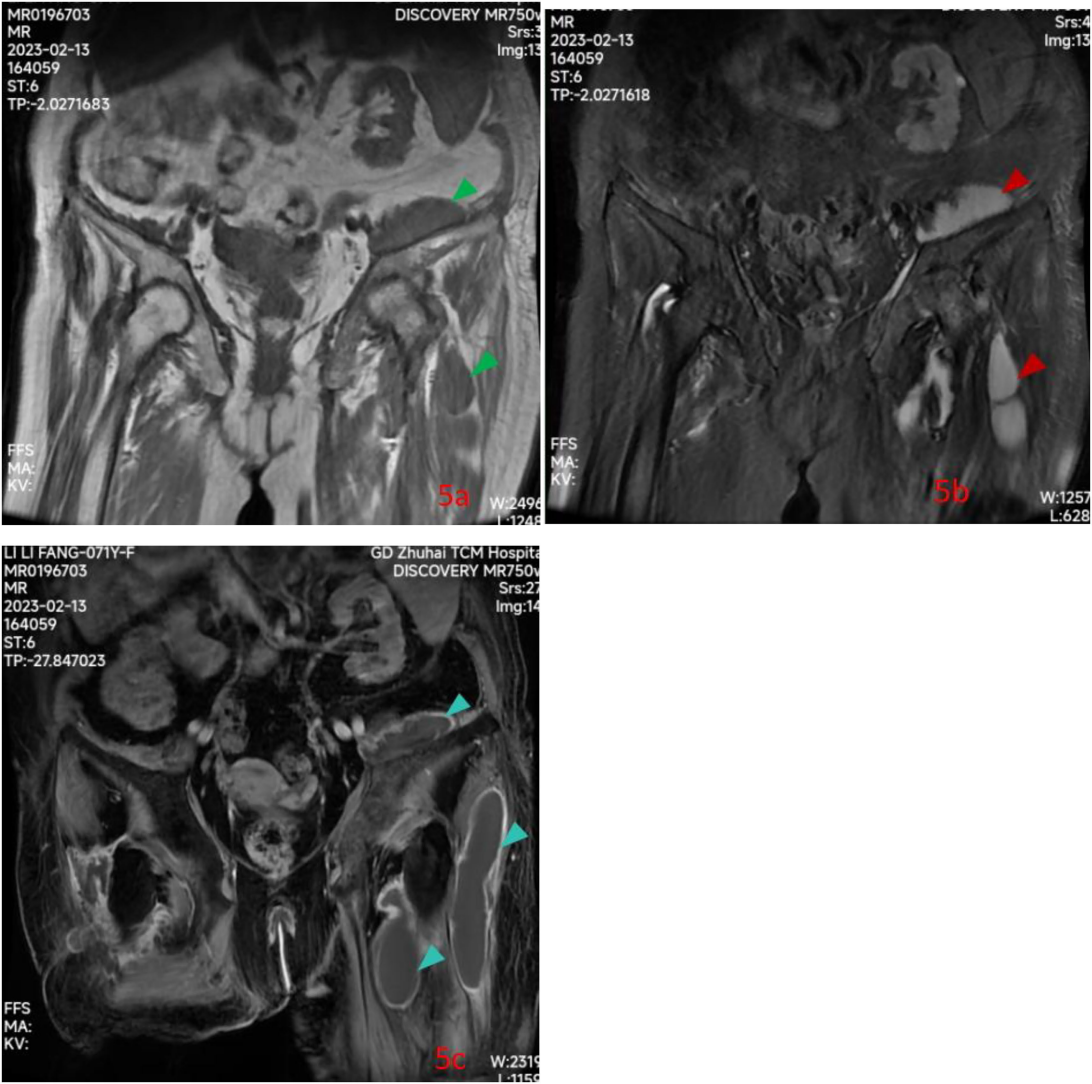
Pelvic contrast-enhanced MRI 10 days after admission showed the cystic lesions were low signal intensity (green triangle) on spin echo T1-weighted images **(a)**, high signal intensity (red triangle) on fat-suppressed fast spin echo proton-density-weighted images **(b)** along the left psoas and iliacus muscles, around left hip; A large hyperdense cystic lesion (royal blue triangle) extending along the iliopsoas muscle and around left hip on enhanced scan images **(c)**.

A 71-year-old woman, unmarried and living alone, was admitted with complaints of pain in the left lower back, fatigue, and difficulty walking; however, she did not present with fever. Her medical history revealed that she had previously been hospitalized elsewhere for approximately 1 month and was treated with systemic anti-infectives, pain relief, and bed rest, without any improvement. She had a history of gout and rheumatoid arthritis for more than 10 years, with poor medical control, but denied any history of diabetes mellitus or tuberculosis. In addition, she denied any history of genetic diseases or trauma. Deformities of right hands and tophi formations in the left finger joints (Figure 1) were noted for more than 10 years. She also possessed a congenital deformity of the posterior spinal processes. On examination, there was moderate tenderness in the left lower quadrant, limited motion of the left hip, and posterolateral swelling of the left hip with an obvious concave scar; however, there was no evidence of a sinus or wound. No fever was observed during her 1st day of admission. Initial laboratory results were as follows: uric acid, 555.9 μmol/L (reference range: 208–428 μmol/L); erythrocyte sedimentation rate (ESR), 113 mm (reference range: 1–15 mm/h); procalcitonin, 0.67; C-reactive protein (CRP), 182.59 mg/L (reference range: 0–5 mg/L); a normal white blood cell count of 8.04 × 109/L with 78.8% neutrophils; hemoglobin, 54 (Hb) g/L; leukocyte count,1.04 × 109/L with 12.9% lymphocytes; platelet count, 221 × 109/L; normal aspartate/alanine aminotransferases, 19.2/13.8 μkat/L; lactate dehydrogenase, 192 μkat/L; low albumin, 26.3 g/L; and deranged kidney function tests, including blood urea nitrogen, 11.9 mmol/L, and serum creatinine, 191 μmol/L. Urinalysis showed nothing abnormal except for urinary latent blood (+). In addition, a stool examination, chest X-ray, ECG, and echocardiographic findings were all normal. Plain pelvic radiographs showed erosion of the sacral and iliac borders, widening of the left sacroiliac joint, condensation of the adjacent bone, joint space narrowing, and a low-density cystic lesion of the left hip (Figure 2). Computed tomography (CT) showed widening of the left sacroiliac joint (Figure 3a) and a multilocular abscess within the left psoas muscle (Figures 3b,c). In addition, juxta-articular soft tissue masses containing sandy calcifications around the pelvis and both hips were also found (Figure 3d). CT findings indicated the possibility of a combined left psoas muscle and left hip abscess, as well as tophaceous gout of both hips. On the afternoon of the 2nd day after admission, the patient developed a high fever of 39.1 °C without chills or other discomfort. After cooling and simple rehydration therapy, the patient’s body temperature quickly returned to normal. On the 3rd day after admission, her temperature remained normal. However, a high fever recurred between approximately 9 p.m. to 11 p.m. over the following 5 days, with the highest temperature reaching 39.3 °C on the 4th day of admission and the lowest 38.8 °C on the 5th day of admission. During the time of fever, she had no chills or discomfort. Body temperature gradually returned to normal after simple physical cooling and fluid replacement. The abnormal temperature lasted for approximately 4 h 1 day. After 5 days, the blood culture results were also normal. Ultrasound-guided aspiration of the cystic lesion in the left hip was performed 3 days after admission. Approximately 100 mL of a milky fluid was aspirated. Additionally, cultures for Gram-positive and Gram-negative bacteria, anaerobic bacteria, and fungi were all negative. Quantitative fluorescence polymerase chain reaction (PCR) of DNA in tuberculosis sputum smears for acid-fast bacteria and fungi was also negative. Anti-TB antibody (Tb-Ab) was also negative; however, the detection of TB infection in the T cells was positive. To better evaluate the pelvic lesion and clarify the diagnosis, a dual-energy CT scan and contrast-enhanced MRI of the pelvis were performed. The dual-energy CT scan showed multiple bilateral gout nodules of the iliac bone and sacrum, as well as the proximal femur (Figure 4). The cystic lesion showed low signal intensity on spin echo T1-weighted images (Figure 5a) and high signal intensity on fat-suppressed fast spin echo proton-density-weighted images of the left psoas and iliacus muscles around the left hip (Figure 5b). In addition, there were Diffusion Weighted Imaging (DWI) signal increases without significantly decreased Apparent diffusion coefficient (ADC) values. Contrast-enhanced MRI revealed a large hyperdense cystic lesion extending along the iliopsoas muscle and around the left hip (Figure 5c). Soft tissue around the lesion showed a slightly higher flocculent T2WI signal with blurred edges. While the MRI helped delineate the extent of the pelvic and left hip abscesses, it could not rule out occult sources of abscess due to bacteria, anaerobic bacteria, fungus, and tuberculosis. On the 2nd day of admission, febuxostat 40 mg was administered orally once a day for the gout treatment; ceftriaxone 2 g intravenous drip was used once a day for anti-infective therapy lasting 8 days, but it did not work as the fever persisted. Then, as a substitute, a levofloxacin 100 mL intravenous drip once a day, rifampicin 0.3 g orally once a day, and diclofenac sodium sustained-release tablet 0.1 g orally once a day were used for fever treatment. There was no fever that night after the change of medication. This medicine lasted for 4 days until the day of surgery. Open surgical intervention was performed under intubation and general anesthesia 12 days after admission using a left para-rectus and posterolateral approach to the hip for effective drainage as well as to avoid further aggravation.

**Figure 6:**
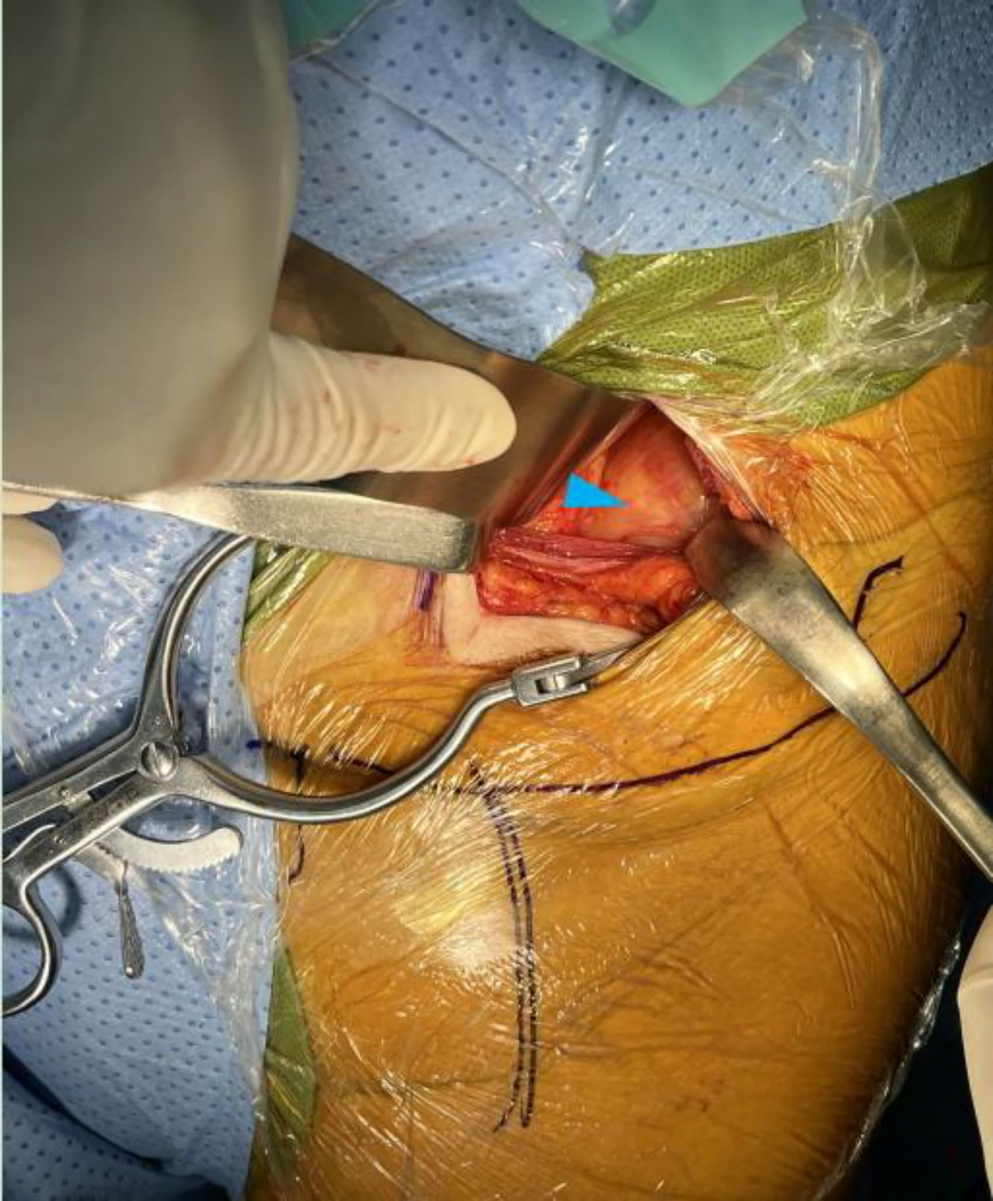
Anterior view of left lower quadrant after laparotomy by para-rectus approach showed cystic mass (blue triangle) in the left iliac fossa intraoperation.

**Figure 7:**
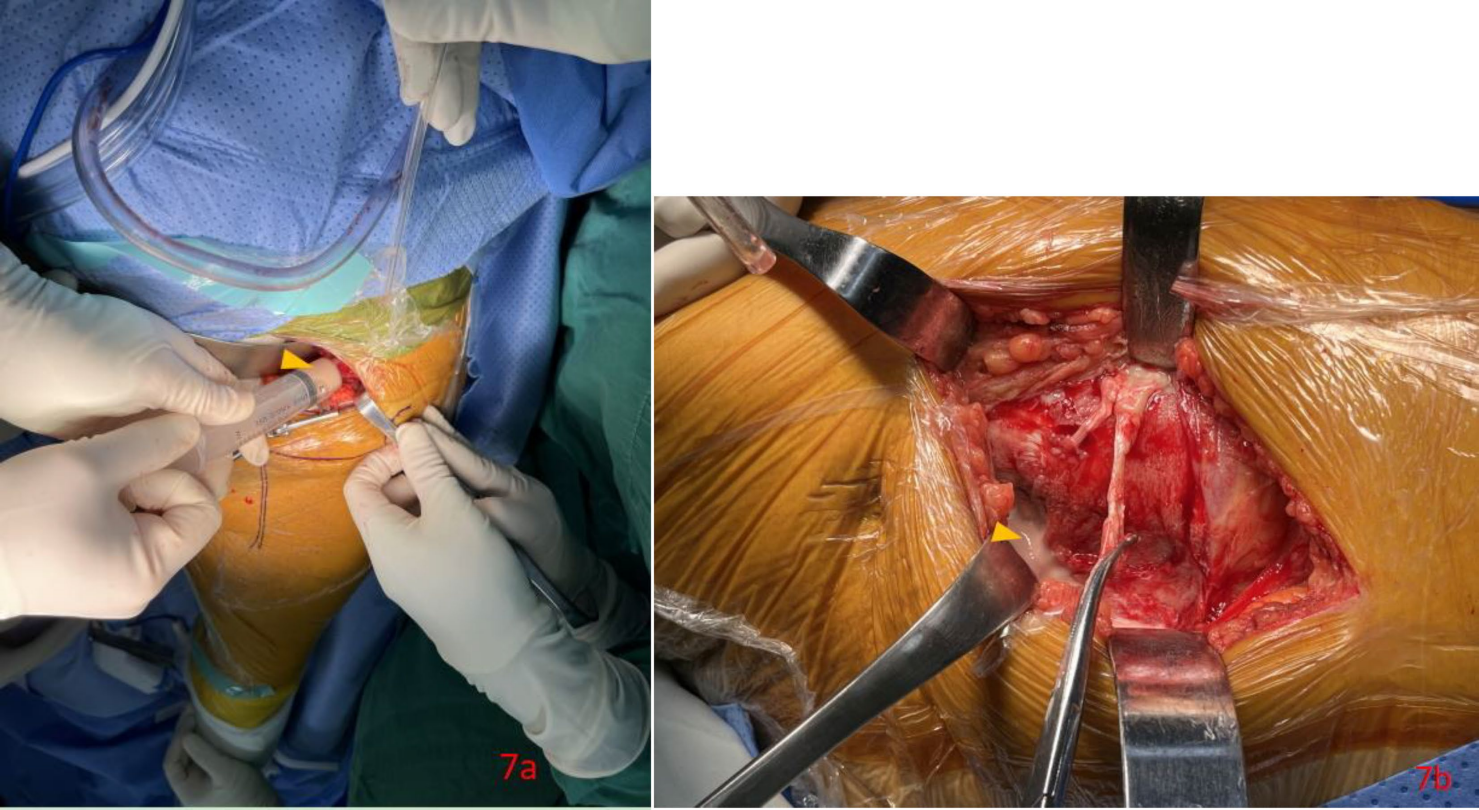
Anterior view of left lower quadrant after laparotomy by para-rectus approach showed milky fluid (orange triangle) was aspirated from cystic mass in the left iliac fossa **(a)** and milky fluid (orange triangle) was found after incision of cystic mass in the left iliac fossa **(b)** intraoperation.

**Figure 8:**
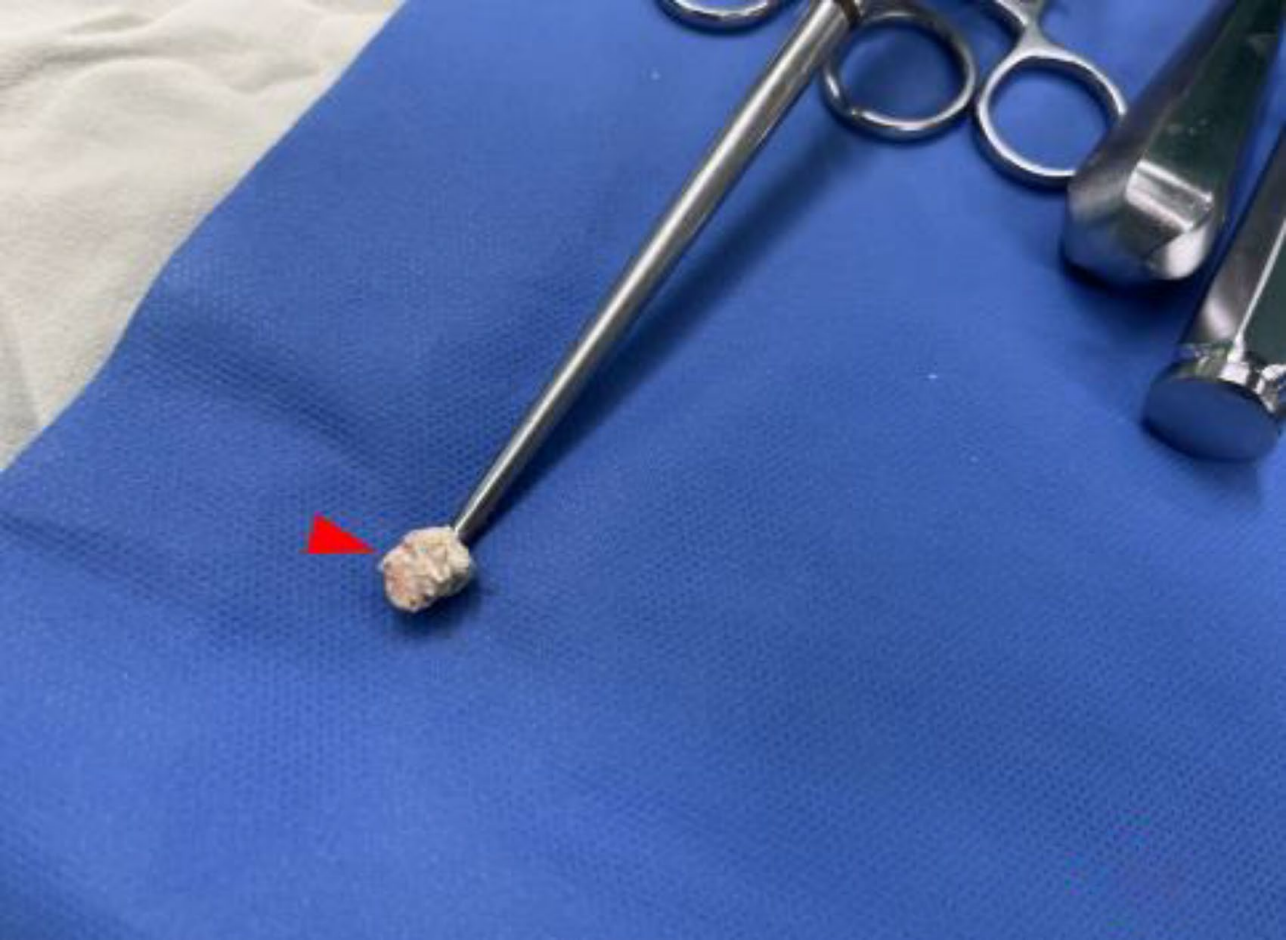
Some milky tophi (red triangle) was showed on curette after scraped from cystic lesion in iliopsoas muscle intraoperation.

The procedure was performed in a floating position. First, a para-rectus approach of the left side was performed in the supine position. The fascia and abdominal peritoneum were separated sequentially by layer, and a cystic mass in the left iliac fossa was identified (Figure 6). Milky fluid was aspirated (Figures 7a,b), and some milky tophi were scraped from the cystic lesion in the iliopsoas muscle (Figure 8). In addition, a large number of tophi-like crystals were found and sent for intraoperative pathology. Following this, the iliopsoas muscle abscess was dissected and thoroughly debrided. The laparotomy incision was closed in the usual manner, with a drainage tube left inside.

**Figure 9:**
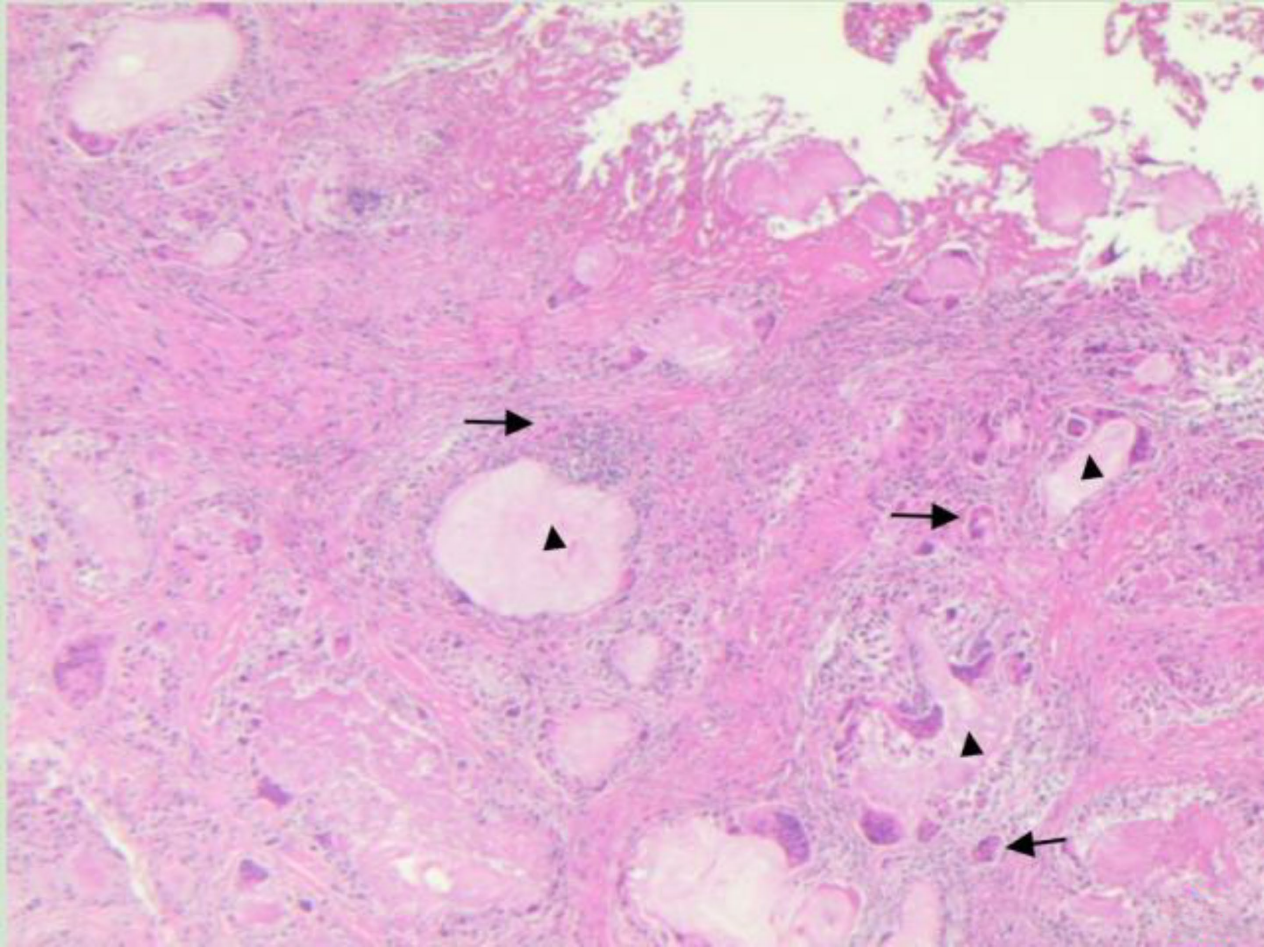
Microscopic morphology of gout nodules show in the fibrous connective tissue, scattered, nodular, amorphous, cell-free reddish substances were observed, namely uric acid crystals (black triangle). Such crystals formed a gout nodule with a foreign body granuloma (black arrow)as the core, surrounded by a layer of macrophages, multinucleated giant cells, scattered lymphocytes and fibroblasts (hematoxylin eosin staining, magnification: 10×).

Subsequently, the left hip abscess was treated in the same manner; however, this was performed in the lateral position, with a 10-cm incision performed via a lateral approach. Intraoperative pathology of these tissues indicated the formation of gout (Figure 9). The patient continued anti-infective medication and gout treatment for 1 week after surgery. While the second intraoperative tissue cultures were positive for Gram-positive and Gram-negative bacteria, tuberculosis and fungal tests were all negative. However, high-throughput gene sequencing of these tissues for various pathogens indicated divergent Mycobacterium tuberculosis without other bacteria, fungi, or anaerobic bacteria.

Levofloxacin 100 mL once a day was continued for 8 days until high-throughput genetic testing confirmed tuberculosis infection 9 days after surgery. An infected psoas abscess due to gout of the iliac psoas muscle, combined with tuberculosis infection, was determined. The intravenous drip antibiotics were stopped. The patient’s anti-tuberculosis regimen changed to isoniazid 0.2 g, pyrazinamide 1 g, ethambutol 0.75 g orally once a day, and rifampin 0.3 g orally twice a day. For gout, she continued febuxostat 40 mg orally and diclofenac sodium sustained-release tablet 0.1 g orally once a day. In the subsequent days, the patient showed significant improvement in her general condition and was discharged from the hospital 10 days post-operation. The patient continued to receive oral anti-tuberculosis and gout medication for 6 weeks. She responded well to the therapy, and the surgical incision healed normally without any discomfort symptoms such as fever or pain in the left hip and left iliac region during the 6-month follow-up. The chronological sequence of key clinical events, diagnostic interventions, and therapeutic management is summarized in Table 1.

**Table 1:** Timeline of diagnostic and therapeutic management.

Time point (day of admission)	Clinical presentation and symptoms	Key diagnostic investigations and results	Interventions and treatment	Outcome and response
Day 1	Diffuse lower back/left hip pain; no fever.	Lab: ↑Uric acid (555.9 μmol/L), ↑CRP (182.59 mg/L), ↑ESR (113 mm/h), severe anemia (Hb 54 g/L), renal impairment. X-ray: erosion/widening of the left sacroiliac joint.	Admission; supportive care.	Baseline established.
Day 2 (Afternoon)	High fever (39.1 °C); no chills.	–	Febuxostat 40 mg/day (for gout) initiated. Ceftriaxone IV (anti-infective) initiated. Physical cooling.	Fever resolved temporarily.
Days 2–7	Recurrent nightly high fever (38.8–39.3 °C); Persistent pain.	Blood cultures (reported day 7): Negative. Ultrasound-guided aspiration (day 3): 100-mL milky fluid. Fluid cultures: Negative for bacteria/fungi. TB tests (sputum smear, PCR, and Tb-Ab): Negative. T-SPOT.TB (TB T-cell test): Positive. Dual-energy CT (day ~5): Confirmed bilateral gout nodules. Contrast-enhanced MRI (day ~5): Large iliopsoas/hip abscess.	Ceftriaxone continued (ineffective). Switch to levofloxacin IV + rifampicin PO + Diclofenac per os (oral) (day 7).	Fever persisted despite initial antibiotics. No fever on the 1st night after medication change.
Day 12	–	Surgery: Open drainage via para-rectus approach. Intraoperative findings: Milky fluid and tophi. Pathology: Confirmed gouty tophus. Tissue cultures: Negative for bacteria/fungi.	Surgical drainage and debridement. Post-operation: Continued levofloxacin and febuxostat.	Successful evacuation of abscess.
Post-operation days 1–8	Afebrile; general condition improving.	High-throughput gene sequencing (mNGS) on intraoperative tissue (reported post-operation day 9): Positive for *Mycobacterium tuberculosis*.	Continued levofloxacin.	Definitive diagnosis of TB co-infection achieved.
Post-operation day 9	–	mNGS result confirmed.	Anti-TB therapy initiated: Isoniazid + rifampin + pyrazinamide + ethambutol. Gout therapy continued: febuxostat.	Targeted treatment commenced.
Post-operation day 10	Well-conditioned; wound healing.	–	Discharged from the hospital.	–
6-month follow-up	No fever or pain; Surgical wound healed.	Clinical review.	Continued oral anti-TB and anti-gout therapy.	Successful recovery with no recurrence.

## Discussion

Gout is the most common crystal-induced arthritis. Aggregates of monosodium urate monohydrate crystals primarily deposit in avascular (e.g., cartilage) or relatively avascular tissues (e.g., tendons and ligaments) (3) around low-temperature peripheral joints and soft tissues (e.g., ears) (2, 5). Tophi deposits in the iliopsoas muscle are extremely rare. Therefore, secondary infections of gouty tophi in the iliopsoas muscle due to tuberculosis are even rarer.

With this particular 71-year-old woman, there were some risk factors for tophi deposition in the iliopsoas muscle. First, uncontrolled gout can lead to polyarticular attacks and tophi deposits in soft tissue and joints (6, 7). The patient did not receive systematic treatment for gout or rheumatoid arthritis, despite a history of longstanding rheumatoid arthritis and gout in her extremities. Additionally, she had concomitant medical diseases, such as chronic renal failure, which was one of several risk factors for tophi deposition (8). Second, her rheumatoid arthritis and chronic renal failure had not been treated systemically, which may have contributed to anemia and poor health. Simultaneously, poor health conditions were one of the risk factors for tuberculosis. Tuberculosis is a wasting disease, which exacerbated her poor conditions. Third, the patient is more than 70 years old. The prevalence of this disease in women tends to approach that observed in men after 60 years of age, as reported in the literature (9).

Dual-energy computed tomography (DECT) is a more recent imaging technique used to establish the diagnosis of gout at uncommon sites (10). A clinical diagnosis for this patient was challenging for several reasons, which may have contributed to a delay. First, it can be difficult to determine whether tophi are infected based on CT or MRI findings alone. However, DECT has a high sensitivity and specificity in identifying MSU crystals of size >3 mm, with an overall accuracy of 87–94% (11). In our case, no wound or ruptured subcutaneous tophi were encountered. The white cell count was normal after admission without specific treatment. Acute stress-associated leukocytosis was considered rather than infection. The classic symptoms of this patient were atypical. These included deep, diffuse pain in the back and hip, which was initially attributed to lumbar intervertebral disc herniation. We found no evidence of secondary infection of the iliopsoas tophi before surgery.

Second, the main clinical signs of this patient were repeated afternoon high fever, diffuse pain in the back and hip, and pain during simple movements that stretch the iliopsoas muscles, which can be confused with primary bacteria-infected psoas abscesses. It is difficult to attribute secondary infection of gouty tophi in the iliopsoas muscle to tuberculosis, even with longstanding gout in the fingers, which was a common daily clinical sign. The patient did not have a history of long-term immunosuppressive therapy, such as corticosteroids, methotrexate, or biologic agents, for her rheumatoid arthritis. Her poor medical control was primarily due to a lack of systematic treatment rather than iatrogenic immunosuppression. However, it is well established that both active rheumatoid arthritis itself and chronic conditions such as renal failure can lead to a state of relative immune dysregulation. This altered immune milieu, coupled with her advanced age and overall debilitated state (as evidenced by severe anemia and hypoalbuminemia), likely created a permissive environment for the reactivation of a latent tuberculous focus. Regarding the source of the infection, the chest X-ray upon admission was unremarkable, and unfortunately, a dedicated chest CT scan was not performed. Therefore, we cannot definitively confirm or rule out the presence of old, healed pulmonary tuberculosis lesions. The patient also explicitly denied any history of invasive physical therapies, such as acupuncture, in the region prior to the onset of her symptoms. In the context of these findings, the most plausible explanation is the reactivation of a latent tuberculous focus. The route of dissemination was likely hematogenous, seeding the pre-existing gouty tophi in the iliopsoas muscle. Gouty deposits can create a unique local inflammatory and micro-environment that may facilitate the survival and proliferation of blood-borne pathogens, including *M. tuberculosis*. This sequence of events—hematogenous spread to a site of pre-existing structural abnormality—is a recognized, though uncommon, pathogenesis for extrapulmonary tuberculosis.

Third, blood cultures during the fever were negative. Cultures of milky fluid intraoperatively aspirated from the cystic lesion in the left hip and soft tissue, as well as cultures of Gram-positive and Gram-negative bacteria, anaerobic bacteria, and fungi, were all negative. Moreover, there was no evidence of tuberculosis. Rather, the pathology of the lesion showed the formation of gouty stones, and high-throughput gene sequencing of intraoperative tissue for various pathogens indicated divergent Mycobacterium tuberculosis more than 2 weeks after admission. This test adopted the metagenomic next-generation sequencing technology based on the Illumina NextSeq 550 platform to conduct unbiased pathogen detection on the lesion tissue samples obtained during the operation. By conducting bioinformatic analysis on the sequencing data and comparing it with a dedicated microbiological database, we specifically detected 182 sequences matching the *Mycobacterium tuberculosis* complex in the tissue samples of this case. These sequences cover the specific genomic regions of *Mycobacterium tuberculosis*, providing molecular evidence for diagnosis. This result contrasts with the negative results of traditional microbial culture and smear, highlighting the unique value of this technique in diagnosing deep tissue infections caused by low biological load or difficult-to-culture pathogens.

The discordance between negative conventional microbiological tests (including acid-fast smear, culture, and PCR) and positive T-cell assay and high-throughput sequencing can be explained by several factors. Localized tuberculous infections in deep musculoskeletal sites often present with a low bacillary load that falls below the detection threshold of smear microscopy and culture. Prior exposure to empirical antibiotics can further suppress bacterial growth, leading to false-negative cultures. The quantitative fluorescence PCR result can be negative due to its limited sensitivity in paucibacillary specimens from closed sites, and the TB antibody test has well-documented low sensitivity and specificity in extrapulmonary tuberculosis. In contrast, the T-cell assay detects a persistent cellular immune response to *M. tuberculosis* antigens and can remain positive even in latent or localized paucibacillary infections. Metagenomic next-generation sequencing (mNGS) is a highly sensitive, culture-independent method capable of detecting minute amounts of microbial DNA. The positive mNGS result for *M. tuberculosis*, despite negative conventional tests, is therefore consistent with a deep, localized tuberculous co-infection where the pathogen burden was too low for conventional methods to detect but sufficient to be identified by the more sensitive molecular techniques.

The diagnostic process for this patient was complex and iterative. The initial differential diagnosis included a primary pyogenic psoas abscess, septic sacroiliitis, spondylodiscitis, and an exacerbation of the patient’s known rheumatic or gouty disease. The diagnostic challenges were multifaceted: the patient’s presentation with non-specific deep pain and persistent fever was atypical for a gout flare, and she failed to respond to broad-spectrum antibiotics, arguing against a common bacterial pathogen. While imaging (CT and MRI) clearly delineated the extensive abscess and raised suspicion for gout, it could not confirm the infectious etiology. The definitive diagnosis was achieved through a stepwise approach: dual-Energy CT objectively confirmed the presence of peri-articular urate crystals, establishing the substrate of gout. The intraoperative pathological findings further corroborated the diagnosis of gouty tophus. Crucially, the failure of conventional microbiological tests (culture, smear, and PCR) to identify a pathogen necessitated the use of mNGS, which provided the definitive evidence of *Mycobacterium tuberculosis* co-infection. This sequence of findings ultimately led to the final diagnosis of a tuberculous superinfection of a pre-existing iliopsoas gouty tophus.

A principal diagnostic challenge in this case was the dissociation between the clinical presentation of a severe, febrile illness and the repeatedly negative conventional microbiological workup. The patient’s history of gout and rheumatoid arthritis initially confounded the clinical picture, as her symptoms could be attributed to a complex flare of her underlying diseases. Furthermore, the radiographic appearance of a multiloculated abscess, indicating an infectious process, is non-specific and can be observed in pyogenic, tuberculous, or even sterile inflammatory conditions. The inability to identify a pathogen through culture, acid-fast smear, or targeted PCR created a significant diagnostic dilemma, delaying the initiation of appropriate therapy.

The prognostic implications of this diagnostic odyssey are significant. A definitive diagnosis was a prerequisite for initiating life-saving and targeted therapy. Without the confirmation of tuberculosis via mNGS, the patient would likely have remained on ineffective broad-spectrum antibiotics, leading to a progression of the abscess, potential systemic sepsis, and irreversible joint and bone destruction. The successful outcome—characterized by resolution of fever, healing of the surgical wound, and the absence of recurrence at six-month follow-up—was directly contingent upon the establishment of the correct diagnosis and the subsequent tailored anti-tuberculous and anti-gout management.

The management of the psoas abscess—of which the cause was not clear—involves the elimination of the abscess and identification of its underlying source (12). It is difficult to successfully treat the patient via intravenous antibiotics purely for her persistently high fever and lack of response to systemic anti-infectives after admission. Image-guided therapeutic percutaneous catheter drainage is becoming more popular and may be successful in the management of some patients (13). However, this treatment was also unlikely to succeed based on the number and size of the abscesses involving the pelvis and hip.

Therefore, open surgical intervention was chosen using a para-rectus approach for effective drainage and to allow easy removal of lesion tissue for high-throughput gene analysis and pathology to confirm the diagnosis. It was the optimal curative treatment method and quite easy based on our available expertise and the number and size of the abscesses. The patient recovered well, with the wound healing normally and the disappearance of the preoperative pain without any fever following anti-gout and anti-tuberculosis treatment.

This case contributes to a very limited body of literature on co-existent gout and tuberculosis in deep muscular locations. A previously reported case by Chen et al. described intra-abdominal gout mimicking an abscess but without a concomitant tuberculous infection (1). Our case demonstrates that the presence of gouty tophi can create a unique nidus for secondary infection, a phenomenon that has been suggested in the literature for common bacterial pathogens but is exceedingly rare for *M. tuberculosis* (14). The diagnostic challenge we encountered aligns with established knowledge that osteoarticular tuberculosis often presents with insidious symptoms and frequently yields negative cultures, leading to delays in diagnosis (15, 16). The superior sensitivity of mNGS over culture in paucibacillary disease has been demonstrated in other forms of extrapulmonary TB, such as tuberculous meningitis and bone infections, and our case further validates its utility in complex musculoskeletal scenarios (17, 18). Finally, the successful surgical outcome via the para-rectus approach is consistent with literature advocating for open drainage and thorough debridement in large, complex, or multiloculated abscesses not amenable to percutaneous drainage (19).

The diagnostic approach in this case highlights both significant strengths and notable limitations. The principal strength was the synergistic use of advanced imaging and modern molecular diagnostics. Dual-energy CT provided a non-invasive and highly specific confirmation of gouty tophi, which was crucial for understanding the underlying pathological substrate. The definitive strength of our approach lay in employing mNGS, which successfully identified *Mycobacterium tuberculosis* in a paucibacillary, culture-negative deep tissue infection where all conventional diagnostic methods had failed. This underscores the invaluable role of mNGS in diagnosing challenging musculoskeletal infections.

However, our approach had several limitations. First, the diagnosis was heavily reliant on a single positive mNGS result. While the detection of 182 unique MTB sequences is strongly indicative of true infection, we acknowledge the theoretical possibility of sample contamination or detection of non-viable organisms, though the clinical context makes this unlikely. The absence of confirmatory culture growth for *M. tuberculosis* remains a limitation, as culture is considered the gold standard for viability and drug susceptibility testing. Second, as a single case report, our findings describe a unique clinical scenario but cannot establish generalizable prevalence or diagnostic protocols. The cost and limited availability of mNGS may also restrict its widespread use in all clinical settings.

## Conclusion

The development of an iliopsoas abscess due to gout of the iliopsoas muscle, combined with tuberculosis infection, is a rare occurrence. Conventional imaging and laboratory tests may not be sufficient to clarify a diagnosis in such unusual cases. For patients with unexplained high fever as the main clinical symptom, when culture and PCR are negative and systemic anti-infection treatment is ineffective, high-throughput gene sequencing for various pathogens may help identify the causative agent and guide targeted treatment. Open surgical intervention using a para-rectus approach can serve as a routine procedure for effective debridement and drainage.

## Data Availability

The original contributions presented in the study are included in the article/supplementary material, further inquiries can be directed to the corresponding author.
